# Crystal structure of 2,5-dimethyl-3-(2-methyl­phenyl­sulfon­yl)-1-benzo­furan

**DOI:** 10.1107/S1600536814022788

**Published:** 2014-10-24

**Authors:** Hong Dae Choi, Uk Lee

**Affiliations:** a Department of Chemistry, Dongeui University, San 24 Kaya-dong, Busanjin-gu, Busan 614-714, Republic of Korea; b Department of Chemistry, Pukyong National University, 599-1 Daeyeon 3-dong, Nam-gu, Busan 608-737, Republic of Korea

**Keywords:** crystal structure, benzo­furan, 2-methyl­phen­yl, sulfon­yl, C—H⋯O hydrogen bonds, C—H⋯π inter­actions, π–π inter­actions

## Abstract

The title compound, C_17_H_16_O_3_S, crystallized with two independent mol­ecules (*A* and *B*) in the asymmetric unit. The dihedral angle between the benzo­furan ring system [r.m.s. deviation of 0.013 (1) for *A* and 0.009 (1) Å for *B*] and the 2-methyl­phenyl ring is 83.88 (5) for *A* and 86.94 (5)° for *B*. In the crystal, the *B* mol­ecules are linked into a chain along the *b-*axis direction by C—H⋯O hydrogen bonds. The *A* mol­ecules are connected on either side of this chain by further C—H⋯O hydrogen bonds. These chains are linked *via* C—H⋯π inter­actions, forming sheets parallel to (100). There are also very weak π–π inter­actions present [centroid–centroid distance = 3.925 (11) Å] involvingthe 2-methyl­phenyl rings of neighbouring *A* and *B* mol­ecules.

## Related literature   

For a related structure and background to benzo­furan deriv­atives, see: Choi & Lee (2014 ▶). For further synthetic details, see: Choi *et al.* (1999 ▶).
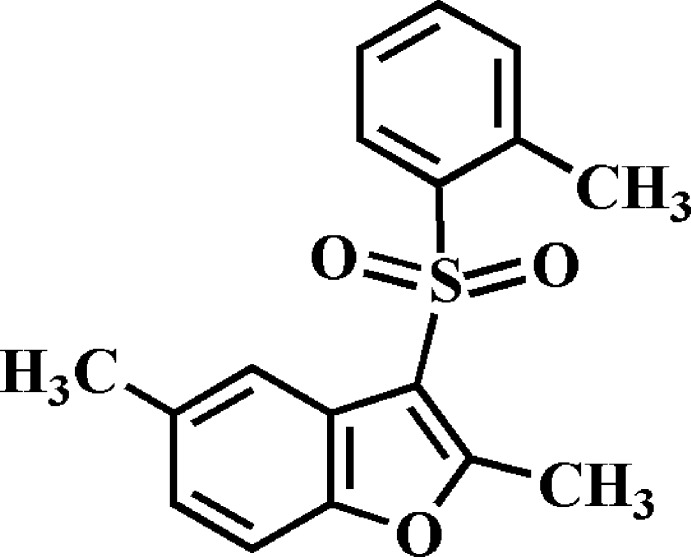


## Experimental   

### Crystal data  

C_17_H_16_O_3_S
*M* *_r_* = 300.36
Monoclinic, 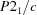

*a* = 16.7338 (4) Å
*b* = 8.0646 (2) Å
*c* = 21.8195 (6) Å
β = 95.296 (1)°
*V* = 2932.00 (13) Å^3^
*Z* = 8
Mo *K*α radiation
μ = 0.23 mm^−1^
*T* = 173 K
0.58 × 0.37 × 0.23 mm

### Data collection  

Bruker SMART APEXII CCD diffractometer
Absorption correction: multi-scan (*SADABS*; Bruker, 2009 ▶) *T*_min_ = 0.879, *T*_max_ = 0.950
27259 measured reflections
6773 independent reflections
5313 reflections with *I* > 2σ(*I*)
*R*_int_ = 0.042

### Refinement  

*R*[*F* ^2^ > 2σ(*F* ^2^)] = 0.043
*wR*(*F* ^2^) = 0.118
*S* = 1.04
6773 reflections
385 parameters
H-atom parameters constrained
Δρ_max_ = 0.34 e Å^−3^
Δρ_min_ = −0.44 e Å^−3^

### 

Data collection: *APEX2* (Bruker, 2009 ▶); cell refinement: *SAINT* (Bruker, 2009 ▶); data reduction: *SAINT*; program(s) used to solve structure: *SHELXS97* (Sheldrick, 2008 ▶); program(s) used to refine structure: *SHELXL97* (Sheldrick, 2008 ▶); molecular graphics: *ORTEP-3 for Windows* (Farrugia, 2012 ▶) and *DIAMOND* (Brandenburg, 1998 ▶); software used to prepare material for publication: *SHELXL97*.

## Supplementary Material

Crystal structure: contains datablock(s) I. DOI: 10.1107/S1600536814022788/su5004sup1.cif

Structure factors: contains datablock(s) I. DOI: 10.1107/S1600536814022788/su5004Isup2.hkl

Supporting information file. DOI: 10.1107/S1600536814022788/su5004Isup3.cml

**Fig. 1.**
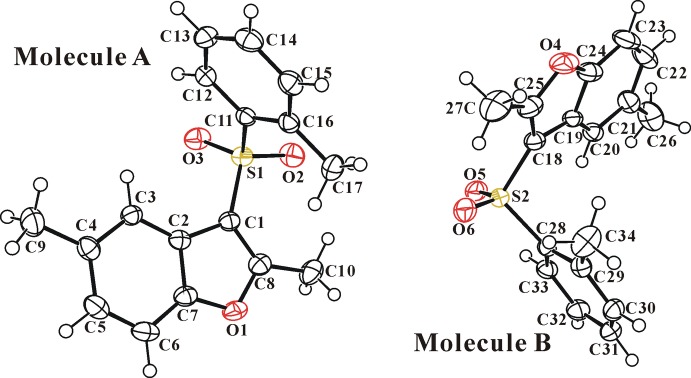
The molecular structure of the two independent molecules (A and B) of the title compound, with atom labelling. Displacement ellipsoids are drawn at the 50% probability level.

. DOI: 10.1107/S1600536814022788/su5004fig1.tif
The mol­ecular structure of the two independent mol­ecules (A and B) of the title compound, with atom labelling. Displacement ellipsoids are drawn at the 50% probability level.

**Fig. 2.**
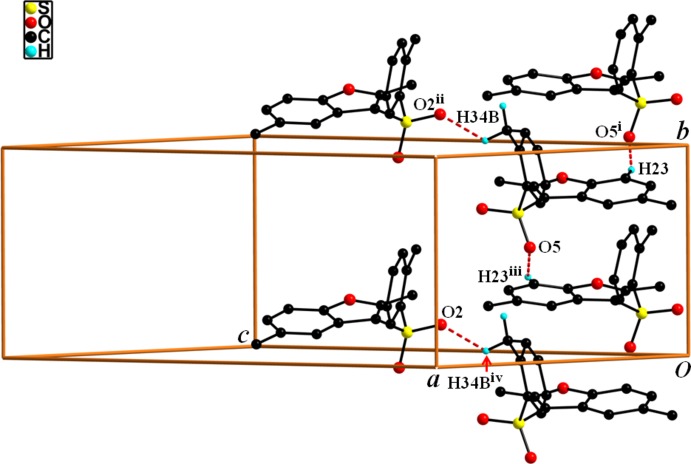
A view of the C—H···O interactions (dashed lines) in the crystal structure of the title compound - see Table 1 for details. H atoms not involved in hydrogen bonding have been omitted for clarity [Symmetry codes: (i) -x, y + 1/2 , -z + 1/2; (ii) x, y + 1, z; (iii) -x, y - 1/2, -z + 1/2; (iv) x, y - 1, z].

Click here for additional data file.x y z x y z x y z x y z . DOI: 10.1107/S1600536814022788/su5004fig2.tif
A view of the C—H⋯O inter­actions (dashed lines) in the crystal structure of the title compound - see Table 1 for details. H atoms not involved in hydrogen bonding have been omitted for clarity [Symmetry codes: (i) −*x*, *y* + 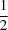, −*z* + 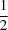; (ii) *x*, *y* + 1, *z*; (iii) −*x*, *y* − 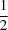, −*z* + 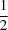; (iv) *x*, *y* − 1, *z*].

**Fig. 3.**
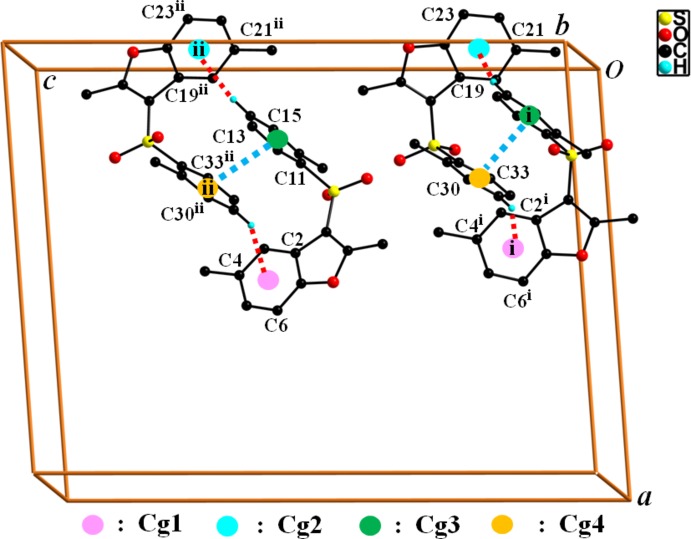
A view of the C—H···π and π–π interactions (dashed lines) in the crystal structure of the title compound - see Table 1 for details. H atoms non-participating in hydrogen-bonding have been omitted for clarity [Symmetry codes: (i) x, -y + 3/2, z - 1/2; (ii) x, -y + 3/2, z + 1/2].

Click here for additional data file.x y z x y z . DOI: 10.1107/S1600536814022788/su5004fig3.tif
A view of the C—H⋯π and π–π inter­actions (dashed lines) in the crystal structure of the title compound - see Table 1 for details. H atoms non-participating in hydrogen-bonding have been omitted for clarity [Symmetry codes: (i) *x*, −*y* + 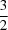, *z* − 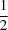; (ii) *x*, −*y* + 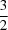, *z* + 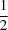].

CCDC reference: 1029621

Additional supporting information:  crystallographic information; 3D view; checkCIF report

## Figures and Tables

**Table 1:** Hydrogen-bond geometry (, ) *Cg*1 and *Cg*2 are the centroids of benzene rings C2C7 and C19C24, respectively.

*D*H*A*	*D*H	H*A*	*D* *A*	*D*H*A*
C23H23O5^i^	0.95	2.52	3.431(3)	162
C34H34*B*O2^ii^	0.98	2.43	3.137(2)	129
C31H31*Cg*1^ii^	0.95	2.93	3.773(3)	148
C14H14*Cg*2^i^	0.95	3.00	3.876(3)	154

Symmetry codes: (i) 
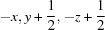
; (ii) 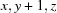.

## supplementary crystallographic information

### S1. Comment

As part of our continuing program on benzofuran derivatives (Choi & Lee, 2014), we report herein on the crystal structure of the title compound which crystallizes with two symmetrically independent molecules (A and B) in the asymmetric unit.

In the title molecules (A and B; Fig. 1), the benzofuran units are essentially planar, with mean deviations of 0.013 (1) and 0.009 (1) Å for A and B, respectively, from the mean planes defined by the nine constituent non-H atoms. The dihedral angles formed by the benzofuran ring systems and the 2-methylphenyl rings are 83.88 (5) and 86.94 (5)° for A and B, respectively.

In the crystal, the B molecules are linked into a chain along the *b* axis direction by C—H···O hydrogen bonds (Table 1 and Fig. 2). The A molecules are connected on either side of this chain by further C-H···O hydrogen bonds (Table 1). These chains are linked via C—H···π interactions forming sheets parallel to (100); see Table 1. There are also very weak π···π interactions present (Fig. 3) involving 2-methylphenyl rings of neighbouring A and B molecules [Cg3···Cg7^i^ = 3.925 (11) Å, where Cg3 and Cg7 are the centroids of rings C11-C16 and C28-C33; symmetry code: (i) = x, -y+3/2, z+1/2].

### S2. Experimental

The starting material 2,5-dimethyl-3-(2-methylphenylsulfanyl)-1-benzofuran was prepared by the literature method (Choi *et al.*, 1999). 3-chloroperoxybenzoic acid (77%, 515 mg, 2.3 mmol) was added in small portions to a stirred solution of 2,5-dimethyl-3-(2-methylphenylsulfanyl)-1-benzofuran (295 mg, 1.1 mmol) in dichloromethane (35 ml) at 273 K. After being stirred at room temperature for 8h, the mixture was washed with saturated sodium bicarbonate solution (2 × 10 ml) and the organic layer was separated, dried over magnesium sulfate, filtered and concentrated at reduced pressure. The residue was purified by column chromatography (hexane-ethyl acetate, 4:1 *v*/*v*) to afford the title compound as a colourless solid [yield 71%; 222 mg; m.p. 385–386 K; *R*_f_ = 0.54 (hexane-ethyl acetate, 4:1 *v*/*v*)]. Single crystals suitable for X-ray diffraction were prepared by slow evaporation of a solution of the title compound (23 mg) in ethyl acetate (20 ml) at room temperature.

### S3. Refinement

All H atoms were positioned geometrically and refined using a riding model, with C—H = 0.95 Å for aryl, and 0.98 Å for methyl H atoms and with *U*_iso_(H) = 1.5*U*_eq_(C) for methyl H atoms and = 1.2*U*_eq_ (C) for other H atoms.

### Crystal data

**Table:**

C_17_H_16_O_3_S	*F*(000) = 1264
*M**_r_* = 300.36	*D*_x_ = 1.361 Mg m^−^^3^
Monoclinic, *P*2_1_/*c*	Melting point = 386–385 K
Hall symbol: -P 2ybc	Mo *K*α radiation, λ = 0.71073 Å
*a* = 16.7338 (4) Å	Cell parameters from 5209 reflections
*b* = 8.0646 (2) Å	θ = 2.3–26.6°
*c* = 21.8195 (6) Å	µ = 0.23 mm^−^^1^
β = 95.296 (1)°	*T* = 173 K
*V* = 2932.00 (13) Å^3^	Block, colourless
*Z* = 8	0.58 × 0.37 × 0.23 mm

### Data collection

**Table:**

Bruker SMART APEXII CCD diffractometer	6773 independent reflections
Radiation source: rotating anode	5313 reflections with *I* > 2σ(*I*)
Graphite multilayer monochromator	*R*_int_ = 0.042
Detector resolution: 10.0 pixels mm^-1^	θ_max_ = 27.6°, θ_min_ = 1.9°
φ and ω scans	*h* = −21→21
Absorption correction: multi-scan (*SADABS*; Bruker, 2009)	*k* = −9→10
*T*_min_ = 0.879, *T*_max_ = 0.950	*l* = −23→28
27259 measured reflections	

### Refinement

**Table:**

Refinement on *F*^2^	Primary atom site location: structure-invariant direct methods
Least-squares matrix: full	Secondary atom site location: difference Fourier map
*R*[*F*^2^ > 2σ(*F*^2^)] = 0.043	Hydrogen site location: difference Fourier map
*wR*(*F*^2^) = 0.118	H-atom parameters constrained
*S* = 1.04	*w* = 1/[σ^2^(*F*_o_^2^) + (0.056*P*)^2^ + 1.1004*P*] where *P* = (*F*_o_^2^ + 2*F*_c_^2^)/3
6773 reflections	(Δ/σ)_max_ = 0.001
385 parameters	Δρ_max_ = 0.34 e Å^−^^3^
0 restraints	Δρ_min_ = −0.44 e Å^−^^3^

### Special details

**Table:**

Experimental. ^1^H NMR (δ p.p.m., CDCl_3_, 400 Hz): 8.25 (d, J=7.52 Hz, 1H), 7.37-7.48 (m, 3H), 7.30 (d, J=8.52 Hz, 1H), 7.26 (d, J=7.52 Hz, 1H), 7.08 (d, J=8.56 Hz, 1H), 2.74 (s, 3H), 2.52 (s, 3H), 2.37 (s, 3H).
Geometry. All esds (except the esd in the dihedral angle between two l.s. planes) are estimated using the full covariance matrix. The cell esds are taken into account individually in the estimation of esds in distances, angles and torsion angles; correlations between esds in cell parameters are only used when they are defined by crystal symmetry. An approximate (isotropic) treatment of cell esds is used for estimating esds involving l.s. planes.
Refinement. Refinement of F^2^ against ALL reflections. The weighted R-factor wR and goodness of fit S are based on F^2^, conventional R-factors R are based on F, with F set to zero for negative F^2^. The threshold expression of F^2^ > 2sigma(F^2^) is used only for calculating R-factors(gt) etc. and is not relevant to the choice of reflections for refinement. R-factors based on F^2^ are statistically about twice as large as those based on F, and R- factors based on ALL data will be even larger.

### Fractional atomic coordinates and isotropic or equivalent isotropic displacement parameters (Å^2^)

**Table:**

	*x*	*y*	*z*	*U*_iso_*/*U*_eq_	
S1	0.29003 (3)	0.10514 (6)	0.48076 (2)	0.02658 (12)	
O3	0.28718 (8)	−0.06396 (16)	0.50124 (6)	0.0361 (3)	
O1	0.51121 (7)	0.27240 (17)	0.48421 (6)	0.0336 (3)	
O2	0.26309 (8)	0.14295 (19)	0.41824 (6)	0.0388 (3)	
C1	0.38848 (10)	0.1730 (2)	0.49575 (8)	0.0257 (4)	
C2	0.43612 (10)	0.1551 (2)	0.55426 (8)	0.0245 (4)	
C3	0.42259 (10)	0.0982 (2)	0.61259 (8)	0.0261 (4)	
H3	0.3718	0.0542	0.6201	0.031*	
C4	0.48413 (11)	0.1065 (2)	0.65957 (9)	0.0306 (4)	
C5	0.55882 (11)	0.1708 (3)	0.64695 (9)	0.0356 (4)	
H5	0.6008	0.1757	0.6794	0.043*	
C6	0.57388 (11)	0.2272 (3)	0.58951 (10)	0.0355 (4)	
H6	0.6249	0.2694	0.5815	0.043*	
C7	0.51090 (10)	0.2191 (2)	0.54429 (8)	0.0285 (4)	
C8	0.43609 (11)	0.2433 (2)	0.45585 (8)	0.0302 (4)	
C9	0.47128 (13)	0.0478 (3)	0.72362 (9)	0.0427 (5)	
H9A	0.4165	0.0746	0.7327	0.064*	
H9B	0.5097	0.1032	0.7535	0.064*	
H9C	0.4794	−0.0724	0.7262	0.064*	
C10	0.42443 (13)	0.2938 (3)	0.39015 (9)	0.0404 (5)	
H10A	0.4554	0.2202	0.3656	0.061*	
H10B	0.4428	0.4083	0.3860	0.061*	
H10C	0.3674	0.2863	0.3756	0.061*	
C11	0.23229 (9)	0.2235 (2)	0.52923 (8)	0.0232 (3)	
C12	0.19580 (10)	0.1353 (2)	0.57375 (8)	0.0283 (4)	
H12	0.2052	0.0196	0.5784	0.034*	
C13	0.14589 (11)	0.2152 (3)	0.61129 (9)	0.0337 (4)	
H13	0.1211	0.1553	0.6419	0.040*	
C14	0.13262 (11)	0.3823 (3)	0.60383 (9)	0.0351 (4)	
H14	0.0978	0.4381	0.6290	0.042*	
C15	0.16958 (11)	0.4699 (2)	0.55985 (9)	0.0330 (4)	
H15	0.1597	0.5856	0.5557	0.040*	
C16	0.22088 (10)	0.3944 (2)	0.52148 (8)	0.0265 (4)	
C17	0.26127 (12)	0.4981 (3)	0.47580 (9)	0.0373 (5)	
H17A	0.2446	0.6140	0.4790	0.056*	
H17B	0.2458	0.4575	0.4341	0.056*	
H17C	0.3196	0.4901	0.4846	0.056*	
S2	0.22038 (3)	0.67798 (6)	0.26437 (2)	0.02693 (12)	
O4	0.00302 (8)	0.83199 (19)	0.28806 (7)	0.0422 (4)	
O5	0.22026 (8)	0.51558 (16)	0.23717 (7)	0.0360 (3)	
O6	0.25599 (8)	0.69703 (19)	0.32615 (6)	0.0404 (4)	
C29	0.27854 (10)	0.9817 (2)	0.22581 (8)	0.0272 (4)	
C18	0.12153 (10)	0.7450 (2)	0.26076 (8)	0.0271 (4)	
C19	0.06680 (10)	0.7498 (2)	0.20564 (8)	0.0263 (4)	
C20	0.07067 (10)	0.7165 (2)	0.14330 (8)	0.0282 (4)	
H20	0.1190	0.6767	0.1289	0.034*	
C21	0.00326 (11)	0.7420 (2)	0.10269 (10)	0.0365 (5)	
C22	−0.06806 (12)	0.7977 (3)	0.12514 (12)	0.0471 (6)	
H22	−0.1141	0.8136	0.0969	0.057*	
C23	−0.07384 (12)	0.8299 (3)	0.18648 (12)	0.0454 (6)	
H23	−0.1225	0.8669	0.2012	0.054*	
C24	−0.00521 (11)	0.8056 (2)	0.22530 (10)	0.0352 (4)	
C25	0.08057 (12)	0.7949 (3)	0.30844 (9)	0.0365 (5)	
C26	0.00614 (15)	0.7143 (3)	0.03466 (10)	0.0534 (6)	
H26A	0.0072	0.8216	0.0137	0.080*	
H26B	−0.0414	0.6520	0.0184	0.080*	
H26C	0.0545	0.6514	0.0276	0.080*	
C27	0.10147 (16)	0.8167 (3)	0.37561 (10)	0.0534 (6)	
H27A	0.0772	0.7273	0.3980	0.080*	
H27B	0.0811	0.9238	0.3886	0.080*	
H27C	0.1599	0.8135	0.3846	0.080*	
C28	0.26882 (10)	0.8114 (2)	0.21492 (8)	0.0235 (4)	
C30	0.32432 (11)	1.0681 (2)	0.18626 (9)	0.0321 (4)	
H30	0.3325	1.1837	0.1924	0.038*	
C31	0.35822 (11)	0.9915 (3)	0.13847 (9)	0.0339 (4)	
H31	0.3903	1.0539	0.1131	0.041*	
C32	0.34591 (11)	0.8247 (3)	0.12726 (9)	0.0345 (4)	
H32	0.3680	0.7725	0.0936	0.041*	
C33	0.30100 (10)	0.7347 (2)	0.16561 (8)	0.0290 (4)	
H33	0.2920	0.6197	0.1583	0.035*	
C34	0.24201 (15)	1.0720 (3)	0.27628 (10)	0.0453 (5)	
H34A	0.2581	1.1889	0.2761	0.068*	
H34B	0.2606	1.0223	0.3160	0.068*	
H34C	0.1834	1.0641	0.2698	0.068*	

### Atomic displacement parameters (Å^2^)

**Table:**

	*U*^11^	*U*^22^	*U*^33^	*U*^12^	*U*^13^	*U*^23^
S1	0.0259 (2)	0.0283 (2)	0.0256 (2)	−0.00190 (17)	0.00290 (17)	−0.00472 (18)
O3	0.0361 (7)	0.0248 (7)	0.0485 (8)	−0.0034 (6)	0.0094 (6)	−0.0059 (6)
O1	0.0275 (6)	0.0383 (8)	0.0366 (7)	−0.0025 (6)	0.0121 (6)	0.0011 (6)
O2	0.0379 (8)	0.0539 (9)	0.0241 (7)	−0.0015 (7)	0.0002 (6)	−0.0076 (6)
C1	0.0246 (8)	0.0262 (9)	0.0268 (9)	0.0009 (7)	0.0055 (7)	−0.0019 (7)
C2	0.0228 (8)	0.0204 (8)	0.0306 (9)	0.0022 (7)	0.0049 (7)	−0.0040 (7)
C3	0.0249 (8)	0.0248 (9)	0.0288 (9)	−0.0013 (7)	0.0041 (7)	0.0009 (7)
C4	0.0302 (9)	0.0274 (9)	0.0335 (10)	0.0026 (7)	−0.0015 (8)	−0.0010 (8)
C5	0.0281 (9)	0.0365 (11)	0.0406 (11)	0.0008 (8)	−0.0059 (8)	−0.0020 (9)
C6	0.0216 (9)	0.0370 (11)	0.0485 (12)	−0.0022 (8)	0.0055 (8)	−0.0028 (9)
C7	0.0257 (9)	0.0272 (9)	0.0337 (10)	0.0018 (7)	0.0085 (7)	−0.0020 (8)
C8	0.0308 (9)	0.0299 (10)	0.0309 (10)	0.0026 (8)	0.0086 (8)	−0.0025 (8)
C9	0.0441 (12)	0.0477 (13)	0.0345 (11)	−0.0005 (10)	−0.0063 (9)	0.0054 (10)
C10	0.0471 (12)	0.0446 (12)	0.0314 (11)	−0.0004 (10)	0.0138 (9)	0.0041 (9)
C11	0.0192 (8)	0.0286 (9)	0.0216 (8)	0.0003 (7)	0.0001 (6)	−0.0013 (7)
C12	0.0248 (9)	0.0302 (10)	0.0294 (9)	0.0003 (7)	0.0006 (7)	0.0031 (8)
C13	0.0291 (9)	0.0438 (12)	0.0292 (10)	−0.0006 (8)	0.0076 (8)	0.0038 (8)
C14	0.0286 (10)	0.0431 (12)	0.0341 (10)	0.0044 (8)	0.0048 (8)	−0.0092 (9)
C15	0.0308 (9)	0.0290 (10)	0.0386 (11)	0.0035 (8)	0.0006 (8)	−0.0033 (8)
C16	0.0235 (8)	0.0279 (9)	0.0275 (9)	−0.0015 (7)	−0.0018 (7)	−0.0006 (7)
C17	0.0418 (11)	0.0302 (10)	0.0401 (11)	−0.0007 (8)	0.0053 (9)	0.0076 (9)
S2	0.0236 (2)	0.0282 (2)	0.0292 (2)	−0.00071 (17)	0.00328 (17)	0.00437 (18)
O4	0.0341 (8)	0.0445 (9)	0.0512 (9)	0.0028 (6)	0.0212 (7)	−0.0050 (7)
O5	0.0304 (7)	0.0246 (7)	0.0543 (9)	0.0007 (5)	0.0106 (6)	0.0034 (6)
O6	0.0377 (8)	0.0534 (9)	0.0292 (7)	−0.0056 (7)	−0.0015 (6)	0.0109 (6)
C29	0.0284 (9)	0.0278 (9)	0.0249 (9)	−0.0003 (7)	−0.0010 (7)	−0.0017 (7)
C18	0.0247 (8)	0.0273 (9)	0.0301 (9)	−0.0007 (7)	0.0074 (7)	−0.0001 (7)
C19	0.0202 (8)	0.0223 (9)	0.0370 (10)	−0.0015 (7)	0.0059 (7)	−0.0001 (7)
C20	0.0236 (8)	0.0244 (9)	0.0366 (10)	−0.0011 (7)	0.0020 (7)	−0.0027 (8)
C21	0.0319 (10)	0.0284 (10)	0.0473 (12)	−0.0030 (8)	−0.0069 (9)	−0.0045 (9)
C22	0.0265 (10)	0.0380 (12)	0.0736 (16)	0.0032 (9)	−0.0128 (10)	−0.0057 (11)
C23	0.0207 (9)	0.0381 (12)	0.0781 (17)	0.0032 (8)	0.0084 (10)	−0.0052 (11)
C24	0.0269 (9)	0.0319 (10)	0.0483 (12)	−0.0003 (8)	0.0118 (9)	−0.0051 (9)
C25	0.0379 (11)	0.0358 (11)	0.0382 (11)	−0.0013 (9)	0.0157 (9)	0.0000 (9)
C26	0.0538 (14)	0.0554 (15)	0.0465 (13)	0.0008 (12)	−0.0190 (11)	−0.0091 (11)
C27	0.0671 (16)	0.0598 (16)	0.0365 (12)	0.0019 (13)	0.0226 (11)	−0.0050 (11)
C28	0.0190 (8)	0.0277 (9)	0.0234 (8)	−0.0006 (7)	0.0003 (6)	0.0015 (7)
C30	0.0334 (10)	0.0281 (10)	0.0339 (10)	−0.0045 (8)	−0.0009 (8)	0.0032 (8)
C31	0.0286 (9)	0.0390 (11)	0.0344 (10)	−0.0030 (8)	0.0050 (8)	0.0095 (8)
C32	0.0306 (10)	0.0407 (11)	0.0336 (10)	0.0030 (8)	0.0108 (8)	−0.0004 (9)
C33	0.0247 (9)	0.0280 (9)	0.0348 (10)	0.0019 (7)	0.0061 (7)	−0.0022 (8)
C34	0.0689 (15)	0.0323 (11)	0.0366 (11)	−0.0067 (10)	0.0157 (11)	−0.0113 (9)

### Geometric parameters (Å, º)

**Table:**

S1—O2	1.4295 (14)	S2—O6	1.4309 (14)
S1—O3	1.4373 (14)	S2—O5	1.4378 (14)
S1—C1	1.7379 (18)	S2—C18	1.7351 (18)
S1—C11	1.7754 (17)	S2—C28	1.7719 (17)
O1—C8	1.369 (2)	O4—C25	1.366 (2)
O1—C7	1.380 (2)	O4—C24	1.380 (2)
C1—C8	1.357 (2)	C29—C30	1.392 (3)
C1—C2	1.449 (2)	C29—C28	1.401 (2)
C2—C7	1.389 (2)	C29—C34	1.497 (3)
C2—C3	1.391 (2)	C18—C25	1.358 (3)
C3—C4	1.386 (2)	C18—C19	1.443 (2)
C3—H3	0.9500	C19—C24	1.391 (2)
C4—C5	1.404 (3)	C19—C20	1.394 (2)
C4—C9	1.510 (3)	C20—C21	1.384 (2)
C5—C6	1.378 (3)	C20—H20	0.9500
C5—H5	0.9500	C21—C22	1.405 (3)
C6—C7	1.377 (3)	C21—C26	1.506 (3)
C6—H6	0.9500	C22—C23	1.376 (3)
C8—C10	1.486 (3)	C22—H22	0.9500
C9—H9A	0.9800	C23—C24	1.377 (3)
C9—H9B	0.9800	C23—H23	0.9500
C9—H9C	0.9800	C25—C27	1.485 (3)
C10—H10A	0.9800	C26—H26A	0.9800
C10—H10B	0.9800	C26—H26B	0.9800
C10—H10C	0.9800	C26—H26C	0.9800
C11—C12	1.390 (2)	C27—H27A	0.9800
C11—C16	1.400 (3)	C27—H27B	0.9800
C12—C13	1.382 (3)	C27—H27C	0.9800
C12—H12	0.9500	C28—C33	1.392 (2)
C13—C14	1.373 (3)	C30—C31	1.378 (3)
C13—H13	0.9500	C30—H30	0.9500
C14—C15	1.383 (3)	C31—C32	1.379 (3)
C14—H14	0.9500	C31—H31	0.9500
C15—C16	1.393 (3)	C32—C33	1.382 (3)
C15—H15	0.9500	C32—H32	0.9500
C16—C17	1.508 (3)	C33—H33	0.9500
C17—H17A	0.9800	C34—H34A	0.9800
C17—H17B	0.9800	C34—H34B	0.9800
C17—H17C	0.9800	C34—H34C	0.9800
			
O2—S1—O3	118.85 (9)	O6—S2—O5	118.11 (9)
O2—S1—C1	108.81 (9)	O6—S2—C18	108.77 (9)
O3—S1—C1	107.34 (8)	O5—S2—C18	107.46 (8)
O2—S1—C11	108.22 (8)	O6—S2—C28	109.53 (8)
O3—S1—C11	106.82 (8)	O5—S2—C28	106.55 (8)
C1—S1—C11	106.09 (8)	C18—S2—C28	105.72 (8)
C8—O1—C7	106.99 (13)	C25—O4—C24	107.11 (14)
C8—C1—C2	107.39 (15)	C30—C29—C28	116.58 (17)
C8—C1—S1	127.81 (14)	C30—C29—C34	119.84 (17)
C2—C1—S1	124.73 (13)	C28—C29—C34	123.57 (17)
C7—C2—C3	119.39 (16)	C25—C18—C19	107.70 (16)
C7—C2—C1	104.81 (16)	C25—C18—S2	127.13 (15)
C3—C2—C1	135.76 (16)	C19—C18—S2	125.11 (13)
C4—C3—C2	119.23 (17)	C24—C19—C20	118.89 (17)
C4—C3—H3	120.4	C24—C19—C18	104.65 (16)
C2—C3—H3	120.4	C20—C19—C18	136.46 (16)
C3—C4—C5	119.14 (18)	C21—C20—C19	119.18 (17)
C3—C4—C9	120.67 (17)	C21—C20—H20	120.4
C5—C4—C9	120.19 (17)	C19—C20—H20	120.4
C6—C5—C4	122.77 (18)	C20—C21—C22	119.5 (2)
C6—C5—H5	118.6	C20—C21—C26	120.71 (19)
C4—C5—H5	118.6	C22—C21—C26	119.77 (19)
C7—C6—C5	116.35 (17)	C23—C22—C21	122.55 (19)
C7—C6—H6	121.8	C23—C22—H22	118.7
C5—C6—H6	121.8	C21—C22—H22	118.7
C6—C7—O1	126.59 (16)	C22—C23—C24	116.24 (19)
C6—C7—C2	123.11 (18)	C22—C23—H23	121.9
O1—C7—C2	110.30 (16)	C24—C23—H23	121.9
C1—C8—O1	110.52 (16)	C23—C24—O4	126.14 (18)
C1—C8—C10	134.49 (18)	C23—C24—C19	123.6 (2)
O1—C8—C10	114.99 (16)	O4—C24—C19	110.24 (17)
C4—C9—H9A	109.5	C18—C25—O4	110.29 (17)
C4—C9—H9B	109.5	C18—C25—C27	134.5 (2)
H9A—C9—H9B	109.5	O4—C25—C27	115.18 (17)
C4—C9—H9C	109.5	C21—C26—H26A	109.5
H9A—C9—H9C	109.5	C21—C26—H26B	109.5
H9B—C9—H9C	109.5	H26A—C26—H26B	109.5
C8—C10—H10A	109.5	C21—C26—H26C	109.5
C8—C10—H10B	109.5	H26A—C26—H26C	109.5
H10A—C10—H10B	109.5	H26B—C26—H26C	109.5
C8—C10—H10C	109.5	C25—C27—H27A	109.5
H10A—C10—H10C	109.5	C25—C27—H27B	109.5
H10B—C10—H10C	109.5	H27A—C27—H27B	109.5
C12—C11—C16	121.63 (16)	C25—C27—H27C	109.5
C12—C11—S1	116.08 (14)	H27A—C27—H27C	109.5
C16—C11—S1	122.22 (13)	H27B—C27—H27C	109.5
C13—C12—C11	120.27 (18)	C33—C28—C29	121.31 (16)
C13—C12—H12	119.9	C33—C28—S2	115.62 (14)
C11—C12—H12	119.9	C29—C28—S2	122.98 (14)
C14—C13—C12	119.17 (18)	C31—C30—C29	122.19 (18)
C14—C13—H13	120.4	C31—C30—H30	118.9
C12—C13—H13	120.4	C29—C30—H30	118.9
C13—C14—C15	120.40 (18)	C30—C31—C32	120.42 (18)
C13—C14—H14	119.8	C30—C31—H31	119.8
C15—C14—H14	119.8	C32—C31—H31	119.8
C14—C15—C16	122.23 (18)	C31—C32—C33	119.08 (18)
C14—C15—H15	118.9	C31—C32—H32	120.5
C16—C15—H15	118.9	C33—C32—H32	120.5
C15—C16—C11	116.27 (17)	C32—C33—C28	120.36 (18)
C15—C16—C17	119.56 (17)	C32—C33—H33	119.8
C11—C16—C17	124.15 (16)	C28—C33—H33	119.8
C16—C17—H17A	109.5	C29—C34—H34A	109.5
C16—C17—H17B	109.5	C29—C34—H34B	109.5
H17A—C17—H17B	109.5	H34A—C34—H34B	109.5
C16—C17—H17C	109.5	C29—C34—H34C	109.5
H17A—C17—H17C	109.5	H34A—C34—H34C	109.5
H17B—C17—H17C	109.5	H34B—C34—H34C	109.5

### Hydrogen-bond geometry (Å, º)

Cg1 and Cg2 are the centroids of benzene rings C2–C7 and C19–C24, respectively.

**Table:**

*D*—H···*A*	*D*—H	H···*A*	*D*···*A*	*D*—H···*A*
C23—H23···O5^i^	0.95	2.52	3.431 (3)	162
C34—H34*B*···O2^ii^	0.98	2.43	3.137 (2)	129
C31—H31···*Cg*1^ii^	0.95	2.93	3.773 (3)	148
C14—H14···*Cg*2^i^	0.95	3.00	3.876 (3)	154

Symmetry codes: (i) −*x*, *y*+1/2, −*z*+1/2; (ii) *x*, *y*+1, *z*.
