# Supplementary figures and images for: Crystal structure of 2,5-dimethyl-3-(2-methyl­phenyl­sulfon­yl)-1-benzo­furan
Source: Acta Crystallogr Sect E Struct Rep Online. 2014 Oct 24;70(Pt 11):o1181–2. doi: 10.1107/S1600536814022788 (PMC4257330; doi:10.1107/S1600536814022788)

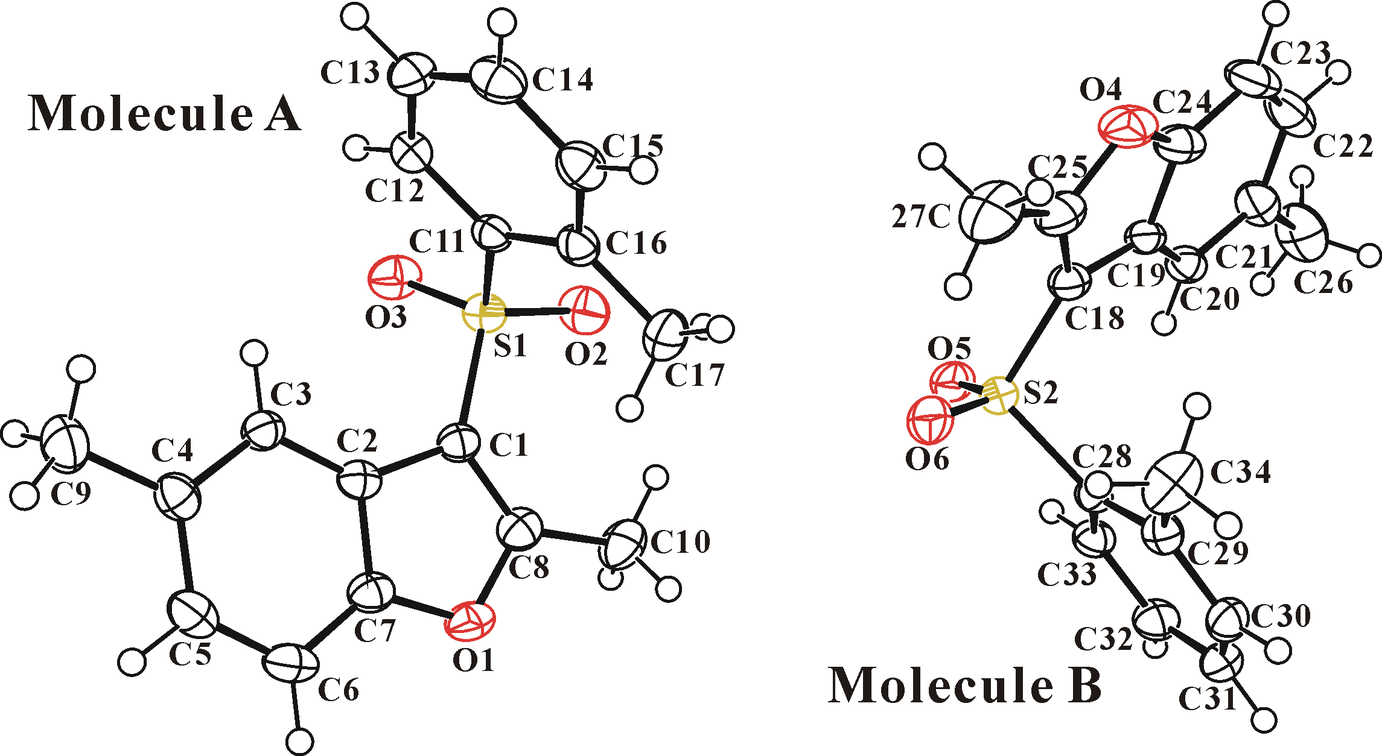

Supplement: Supplementary file 4 [file e-70-o1181-fig1.tif]

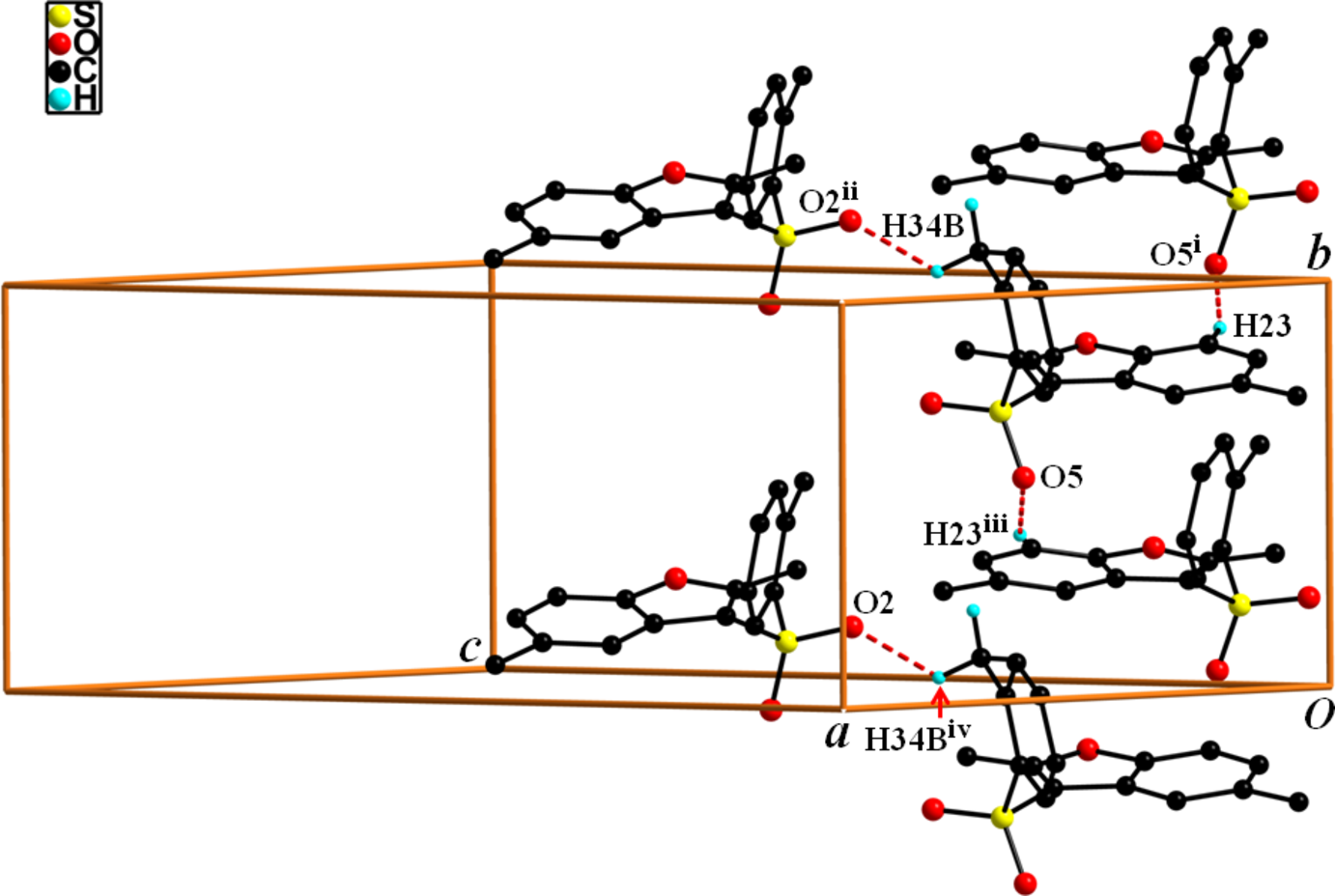

Supplement: Supplementary file 5 [file e-70-o1181-fig2.tif]

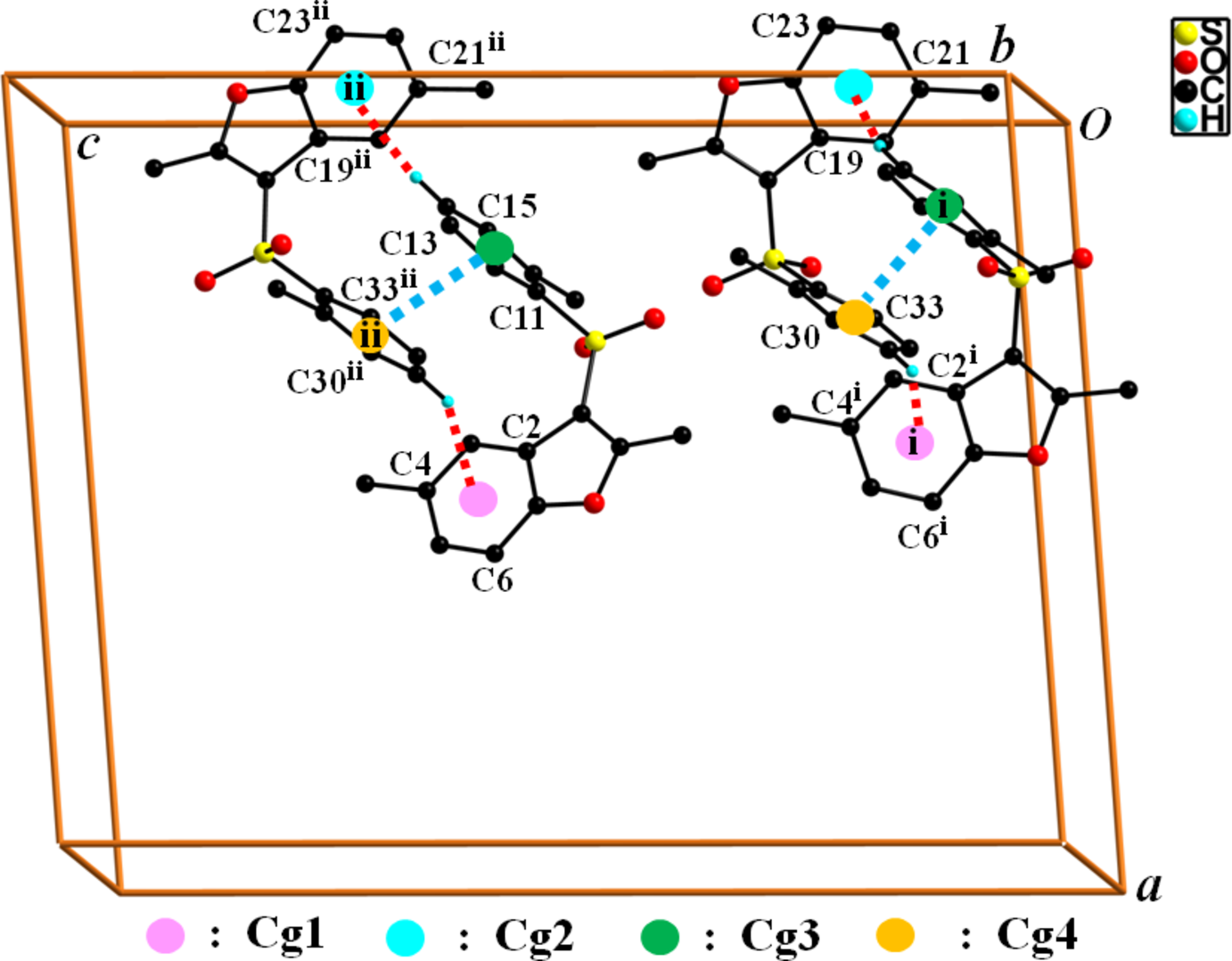

Supplement: Supplementary file 6 [file e-70-o1181-fig3.tif]
